# The Effect of 3-Thiopheneacetic Acid in the Polymerization of a Conductive Electrotextile for Use in Biosensor Development

**DOI:** 10.3390/bios3030286

**Published:** 2013-07-29

**Authors:** Shannon K. McGraw, Evangelyn Alocilja, Andre Senecal, Kris Senecal

**Affiliations:** 1 Biosystems and Agricultural Engineering, Michigan State University, 524 S. Shaw Lane, 115 Farrall Hall, East Lansing, MI 48824, USA; E-Mail: shannon.k.mcgraw2.civ@mail.mil; 2 Food Protection Team, U.S. Army Natick Soldier Research, Development, and Engineering Center (NSRDEC), Natick, MA 01760, USA; E-Mail: andre.g.senecal.civ@mail.mil; 3 Macromolecular Sciences and Engineering Team, U.S. Army NSRDEC, Natick, MA 01760, USA; E-Mail: kris.j.senecal.civ@mail.mil

**Keywords:** 3-thiopheneacetic acid, electrotextile, biosensor, polypyrrole, antibody attachment

## Abstract

Investigations were conducted to develop an electrotextile using a nonwoven polypropylene fiber platform conformally coated in a conductive, functionalized copolymer of polypyrrole and 3-thiopheneacetic acid (3TAA). The objectives of this study were to determine: (1) if the inclusion of 3TAA in the polymerization process would have an effect on the availability of binding sites in the high-surface area electrotextile for biorecognition elements and (2) how the increase in the concentration of 3TAA would affect the physical characteristics of the coating, resistivity of the sample and availability of binding sites. It was found that the addition of 3TAA to the polymerization process resulted in an increase in the size of the polypyrrole coating, as well as the material resistivity and available binding sites for biorecognition elements. These factors were used to determine which of the tested concentrations was best for biosensor development. A polymer coated membrane sample containing a concentration within the range of 10–50 mg/mL of 3TAA was selected as the best for future biosensor work.

## 1. Introduction

Immuno-sensors utilize pathogen-specific antibodies coupled to a transducer as the biological recognition element for detection [1]. The benefits of the antibody-antigen reaction are well known, including high binding efficiency and specificity of detection. In addition, antibodies have been generated against a wide array of bacterial pathogens. Immuno-sensors have been shown to exhibit faster speed and lower cost compared to standard culture detection methods and DNA-based biorecognition techniques, making them especially marketable to the food industry [2,3,4].

In an electrochemical biosensor, the biological recognition element is immobilized on an electrode, which then converts the biological recognition event (*i.e*., antibody-antigen binding) into a measurable electrical signal [1]. One of the benefits of electrochemical impedance-based sensing is that it does not require enzyme labels or redox mediators to facilitate detection as optical-based sensing does [5]. In electrochemical impedance-based systems, a measurable system response is created when the biological recognition event disrupts the flow of the current at the working electrode, while the reference electrode maintains a constant potential [1].

A new field in the development of electrochemical-based biosensors is being explored using high-surface area electrospun membranes. These membranes have been shown to be versatile and can be developed into electrotextile “smart membranes” designed for use with all forms of sensor signal transduction. Previous work has been done to develop electrically active non-metallic textile coatings made of doped polypyrrole polymers [6,7,8,9]. By producing a conductive polymer coating on non-woven microfibers, an electrochemical biosensor electrode can be created that is less expensive than its planar metal counterpart [10], with more available surface area. These electrotextile electrodes can be engineered to emphasize qualities important in biosensor development: durability, disposability and the need for minimal attachment chemistry. The ability to use antibody functionalized fibers for capture, concentration and detection was previously demonstrated with electrospun nanofibers and a carboxyl functional group [11,12,13,14]. With the attachment of biological recognition elements to the electrotextile surface, these electrodes have the capacity to perform pathogen capture, concentration and detection. This would simplify a food pathogen biosensor, resulting in a significantly smaller and lighter detection system. The inclusion of 3-thiopheneacetic acid (3TAA) in the polymerization of such an electrotextile would provide the needed functional group sites for the binding of biorecognition elements necessary to a biosensor design (e.g., antibodies, avidin). Previous work has demonstrated the successful copolymerization of pyrrole and 3TAA to make a functionalized conductive polymer coating, but has not explored the various effects of that inclusion on multiple characteristics of the polymer [11,13,14,15].

The objectives of this study were to determine the following: if the inclusion of 3TAA in the polymerization process would have an effect on the availability of binding sites in a high-surface area electrotextile for biorecognition elements; and how the increase in the concentration of 3TAA would affect the physical characteristics of the coating, resistivity of the sample and available number of binding sites. These factors were used to determine which of the tested concentrations was best for biosensor applications.

## 2. Experimental

### 2.1. Materials

Nonwoven polypropylene microfibers were obtained from North Carolina State Nonwovens Cooperative Research Institute. The fibers were formed into a spunbond fabric with regular periodic spot-melt points to create structural integrity across the fabric. The fibers were cut into circular discs with a diameter of 1.2 cm. They were coated with a doped conductive polypyrrole polymer. For the polymer synthesis, the monomer used was a 10% (v/v) pyrrole solution that was copolymerized with carboxylic acid functional 3TAA. The oxidant was iron (III) chloride (FeCl_3_). The polymer was doped using 5-sulfosalicylic acid (5SSA). Water was used as the reaction solvent. All of the polymerization chemicals were obtained from Sigma-Aldrich (St. Louis, MO, USA). Covalent attachment of fluorescein isothiocyanate (FITC) labeled avidin (Thermo Fisher Scientific, Waltham, MA, USA) was performed using N-(3-dimethylaminopropyl)-N’-ethylcarbodiimide hydrochloride (EDC) (Sigma-Aldrich) and N-hydroxysulfosuccinimide (sulfo-NHS) (Invitrogen, Carlsbad, CA, USA) with 50 mM 2-(N-morpholino)ethanesulfonic acid (MES) buffer, pH 6.0 (Thermo Fisher Scientific).

### 2.2. Synthesis

A previously published aqueous deposition process for the conductive and functional polymer coatings upon a polypropylene fiber matrix was used [11,14]. Polypropylene microfiber mats were briefly submerged in a solution of 10% pyrrole, 90% water and varying concentrations of 3TAA (concentrations of 0, 1, 10, 20, 50 or 100 mg/mL). The functionalized monomer was absorbed onto the fiber mat. The wet fiber sample was then removed from the solution and placed in a glass container for polymerization. FeCl_3_ (0.1 M, 10 mL) was added to the sample to initiate the chemical reaction, while a dopant, 5SSA (0.1 M, 1 mL), was simultaneously added. The fibers in solution were incubated at room temperature for 30 min with constant agitation, thereby ensuring that polymerization occurred on both sides of the mat. The nonwoven fiber sample was removed from the solution, gently rinsed on both sides with deionized (DI) water and dried at room temperature overnight.

### 2.3. Characterizations

Five different methods were used to analyze the effect of the concentration of 3TAA: scanning electron microscopy (SEM) was conducted for a visual assessment, energy dispersive spectroscopy (EDS) was used to determine elemental weight percentages, Fourier transform infrared spectroscopy (FTIR) was used to monitor changes in the C–O stretching band for the carboxylate unit, resistivity measurements were taken across a cross section of the fiber membranes and fluorescence measurements were taken after FITC-avidin was covalently bound to the functional sites.

#### 2.3.1. Scanning Electron Microscopy and Energy Dispersive Spectroscopy

A visual assessment was conducted using scanning electron microscopy. Energy dispersive spectroscopy was used to determine elemental weight percentages. The samples were gold sputter coated and imaged with a Zeiss EVO 60 scanning electron microscope fitted with an EDS attachment (Carl Zeiss Microscopy, LLC, Thornwood, NY, USA). Pictures of each sample were taken at 100× and 5,000× magnification. EDS measurements were performed with 102.4 µs amp time for 500 counts at magnifications of 100×.

#### 2.3.2. FTIR Analysis

FTIR spectra for the samples were obtained by measuring the coated fiber membranes using a Nicolet 6700 FT-IR Spectrometer (Thermo Scientific, Lanham, MD, USA). Triplicate readings were taken for each sample and averaged to obtain the sample spectrum.

#### 2.3.3. Electrical Resistivity

Resistivity measurements were taken of the fiber membranes using a four point probe (Pro-4, Signatone, Gilroy, CA, USA) and a Keithley 2400 Sourcemeter (Keithley Instruments, Cleveland, OH, USA). Triplicate readings of 3 samples per concentration of 3TAA for a total of 9 measurements per sample were taken for each sample. Dixon’s Q test was used to identify and reject outliers using a 99% confidence level. The measured resistivity values were then averaged to obtain the reported sample resistivities.

#### 2.3.4. Avidin Attachment and Fluorescent Output

Fluorescein isothiocyanate labeled avidin (FITC-avidin) was attached to the functionalized membranes through EDC/Sulfo-NHS crosslinking. The discs were washed with DI water and left to dry for 10 min at room temperature. A volume of 200 µL of EDC and Sulfo-NHS in MES buffer was added to each disc and left to react with gentle agitation for 15 min. The discs were then washed twice with MES buffer. A volume of 250 µL of FITC-avidin was added to each disc and reacted with gentle agitation for 4 h. The discs were washed with MES buffer and, then, washed in triplicate with phosphate buffered saline (PBS). The samples were read using a Fluoroskan Ascent microplate fluorometer (Thermo Scientific) and measured for fluorescence at an excitation wavelength of 490 nm. Emission was measured at 535 nm. Triplicate readings were taken for each sample and averaged to obtain an average fluorescent output value.

## 3. Results and Discussion

### 3.1. Results

Increases in resistivity, sulfur weight percent, the presence of carboxyl groups and fluorescent output were all observed as the concentration of 3TAA increased in the samples. These increases became apparent at a concentration of 10 mg/mL of 3TAA in the monomer solution. A summary of results can be found in Table 1.

**Table 1:** Characterization of polypyrrole copolymer with increasing concentrations of 3-thiopheneacetic acid (3TAA) (resistivity and fluorescent output are averages ± the standard error of the mean). RFU: relative fluorescence unit.

Concentration of 3TAA(mg/mL)	Average Resistivity(Ω∙cm)	Sulfur Weight(%)	Average Fluorescent Output(RFU)
0	4.6 ± 0.4	0.93	1.0287 ± 0.0205
1	3.4 ± 0.1	0.55	1.3870 ± 0.1344
10	6.3 ± 0.3	1.30	1.4770 ± 0.1875
20	7.6 ± 0.2	1.54	1.2677 ± 0.1071
50	9.4 ± 0.6	2.24	1.6453 ± 0.2408
100	1,587.4 ± 429.9	3.83	3.9623 ± 1.3675

#### 3.1.1. SEM Analysis

**Figure 1:**
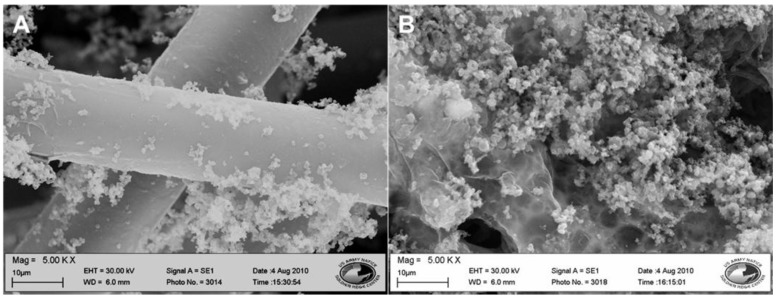
SEM images of fibers with polymer coating at 5,000× magnification. (**A**) 3TAA concentration of 10 mg/mL. A smooth conformal polymer coating was observed along the individual fibers with minimal polymer clusters. (**B**) 3TAA concentration of 100 mg/mL. The coating is rough, with a large amount of polymer built up along the surface, engulfing several fibers and reducing the porosity of the membrane.

The increase in concentration of 3TAA in the polymerization process resulted in an increase in the buildup of the coating on the polypropylene fibers. Little visible difference was observed between the samples ranging in concentration from 0–10 mg/mL. The four samples tested within this range showed a conformal polymer coating around the individual polypropylene fibers. Along the fibers, small buildup of polymer could be observed. An example of this can be seen in Figure 1(A), where fibers were coated with a concentration of 10 mg/mL. Samples with higher 3TAA concentration displayed large buildups of polymer that had collected together to form aggregates measuring roughly 400–500 µm in diameter on the fiber surface. As seen in Figure 1(B), fibers coated at a concentration of 100 mg/mL show polymer build up along the surface, engulfing several fibers and reducing the porosity of the membrane instead of forming a smooth conformal polymer coating along the individual fibers. It was also observed that as the concentration of 3TAA was increased, the polymer coating became more brittle. Flakes of polymer fell off of the samples containing 50 and 100 mg/mL when handled.

#### 3.1.2. Electrical Resistivity

**Figure 2:**
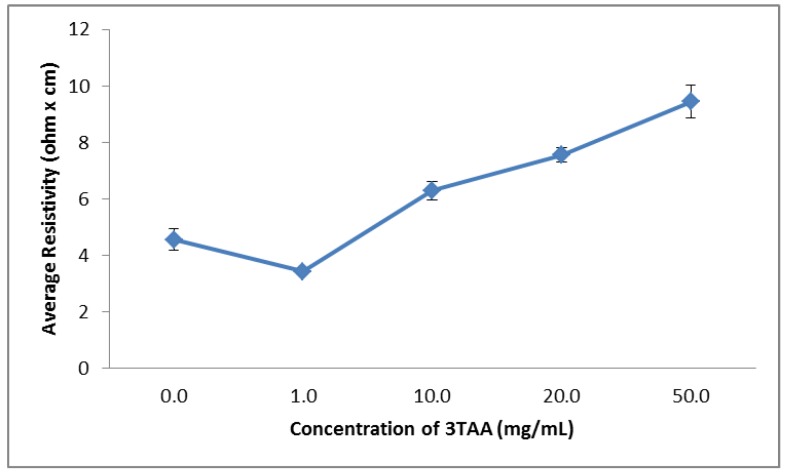
Change in sample resistivity based on increasing 3TAA concentrations, with error bars representing the standard error of the mean of each sample. The overall trend shows resistivity increasing as the concentration of 3TAA increases, starting at 1 mg/mL. The measured value for a concentration of 100 mg/mL has been excluded, due to a difference in scale, so that small changes among lower concentrations may be observed.

As can be seen in Table 1, the measured average resistivities of the samples range from 3.4 to 1,587.4 Ω·cm. The samples containing 0, 1, 10, 20 and 50 mg/mL of 3TAA all have resistivity’s under 10 Ω·cm, with the samples composed from 0 and 1 mg/mL under 5 Ω·cm. A sharp increase is observed in the resistivities of the samples containing 100 mg/mL of 3TAA, with the average resistivity being over 150× larger than the sample containing 50 mg/mL. We hypothesize that at this concentration of 3TAA, self-polymerization occurs between the 3TAA molecules. Because the 3TAA is the non-conductive component of the coating, this results in the disproportional increase in material resistivity. A steady increase in average resistivity is observed as the concentration increased, starting at 1 mg/mL. A Student’s *t*-test (two tails, α = 0.05) shows a significant difference between all samples. The relationship between the concentration of 3TAA in each sample and the resistivity of the sample can be seen in Figure 2. The results in Figure 2 do not include the results for a concentration of 100 mg/mL of 3TAA, so that small changes between the lower concentrations could be observed.

#### 3.1.3. Elemental Weight Percent

Because the chemical structure of 3TAA contains a free carboxyl attached to a sulfur ring, the presence of sulfur was used as an indicator of the presence of carboxyl groups in the coating surface for covalent binding. Energy dispersive spectroscopy was used to determine the elemental weight percentages for each sample. 

**Figure 3:**
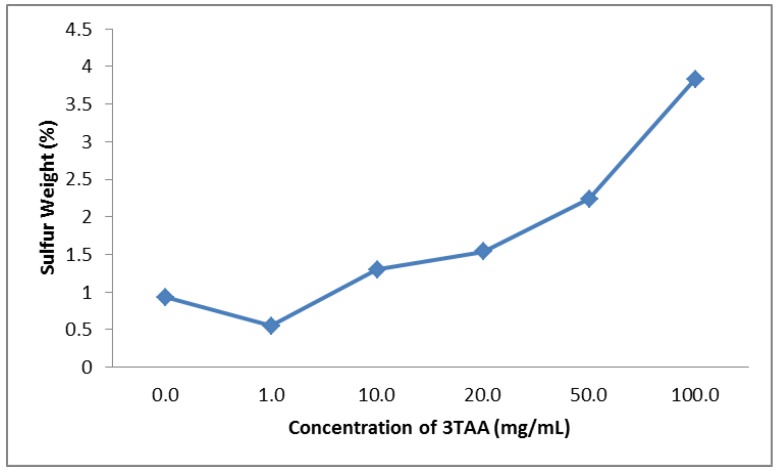
Change in sulfur weight percent at 100× magnification using energy dispersive spectroscopy (EDS) based on increasing 3TAA concentrations. The overall trend shows the sulfur weight percentage increasing as the concentration of 3TAA increases in the sample, starting at 1 mg/mL.

The sulfur weight percent measured in each sample can be seen in Table 1, ranging from 0.55% to 3.83%. All of the samples with 3TAA concentrations of 10 mg/mL or higher have a sulfur weight percent of greater than 1%. The measured sulfur weight percent decreases by 0.38% between 0 and 1 mg/mL. The range between the measurements of the samples containing 10 and 20 mg/mL of 3TAA is 0.24%. A sharp increase in the weight percent of sulfur is observed between the samples containing 50 and 100 mg/mL of 3TAA with a range of 1.59%. Because sulfur is also present in the dopant, 5SSA, only changes in the sulfur weight percent from the sample containing 0 mg/mL 3TAA can be attributed to the 3TAA presence. The relationship between the weight percent of sulfur and the concentration of 3TAA in each sample can be seen in Figure 3.

#### 3.1.4. FTIR

**Figure 4:**
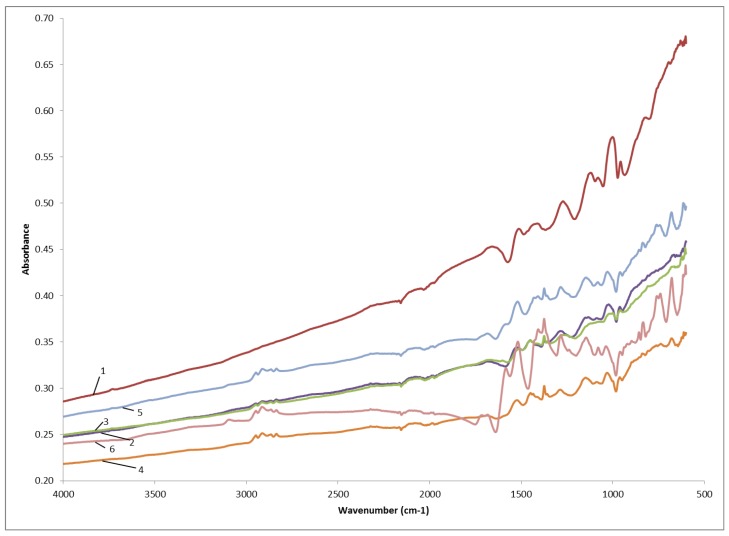
Averaged FTIR spectra for polypyrrole coating with the addition of various concentrations of 3TAA: 0 mg/mL (1); 1 mg/mL (2); 10 mg/mL (3); 20 mg/mL (4); 50 mg/mL (5); 100 mg/mL (6).

Because EDS is morphology-dependent, it is not generally considered a quantitative technique. In order to support our findings about the increased presence of carboxyl groups in the polymer, due to the increase of 3TAA concentration in the monomer, FTIR spectra for each of the tested sample concentrations were generated. Because of variability in the thickness of the coatings, different absorption rates were observed. As the concentration of 3TAA increases, the C–S–C peak (~ 670 cm^–1^) showing planar deformation of the thiophene ring becomes more pronounced. A peak is also observed at a wavelength of 750 cm^–1^, possibly corresponding to the C–H group being out of plane mode in the thiophene ring. An increase in the C–C stretching of the thiophene ring (~1,370 cm^–1^) is also observed as the concentration of 3TAA increases. Peaks not found in pure pyrrole are also observed at wavelengths of 1,500 cm^–1^, between 1,550 and 1,700 cm^–1^ and at 2,900 cm^–1^ and appear to increase at increasing concentrations of 3TAA. The increases observed between 1,550 and 1,700 cm^–1^ are particularly notable, because they are most likely due to the C=O stretching for the acetic acid in the 3TAA. These peaks are first seen at a concentration of 10 mg/mL, but become pronounced at 50 mg/mL. This data indicates that the 3TAA is copolymerizing with the pyrrole during the aqueous deposition polymerization onto the polypropylene microfibers. These results can be seen in Figure 4.

#### 3.1.5. Fluorescence

**Figure 5:**
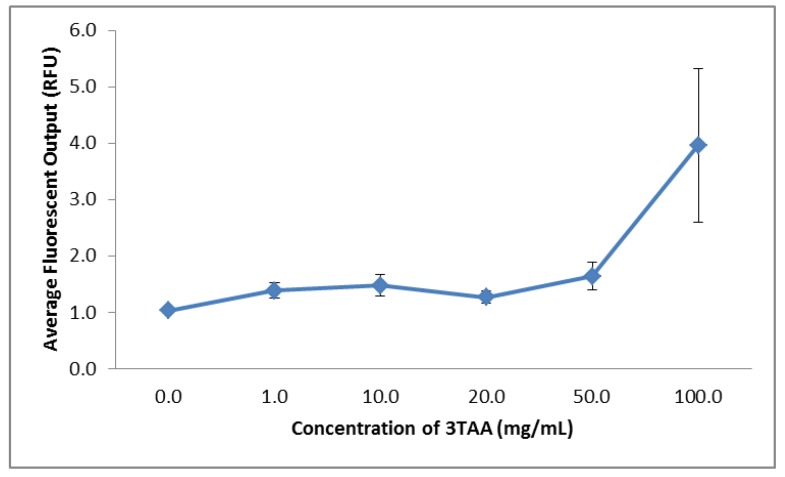
Change in average fluorescent output after FITC-avidin binding based on increasing 3TAA concentrations with error bars showing the standard error of the mean for each sample. The overall trend shows the average fluorescence output increasing at larger concentrations of 3TAA.

The intensity of the FITC signal measured following the crosslinking reaction was used as an indicator of the relative amount of avidin that was successfully attached to the available binding sites provided by the presence of carboxyl groups in the polymer coating. The average fluorescence output for each sample can be seen in Table 1. The average fluorescence signal measured range from 1.0287 to 3.9623 relative fluorescence units (RFUs). Only the sample containing 0 mg/mL of 3TAA measures below the value of 1.1 RFUs. Although the average fluorescent output of the sample containing 0 mg/mL 3TAA was the lowest of all samples measured, there was still an unexpected fluorescent signal. This is most likely due to the FITC-avidin nonspecifically attaching to the rough surface and in the pores between the polymer coated fibers. The samples containing 50 and 100 mg/mL both exceed 1.5 RFUs. The sharpest increase in signal comes between the samples containing 50 and 100 mg/mL, with the difference being 2.317 RFUs. The increase in concentration of 3TAA in each sample coincides with an increase in fluorescent signal for every sample, except between 10 and 20 mg/mL. The relationship between the average fluorescent readout value and concentration of 3TAA in each sample can be seen in Figure 5.

### 3.2. Discussion

The purpose of this study was to determine if the inclusion of 3TAA in the polymerization process would have an effect on the availability of binding sites for bio recognition elements; how the increase in the concentration of 3TAA would affect the physical characteristics of the coating, resistivity of the sample and number of binding sites available; and to determine which concentrations would be best for immuno-sensor development. In all five characterization methods used, a difference was observed between samples that did and did not include 3TAA. The trend was observed for each characterization that after an initial threshold was met, the measured difference increased as the concentration of 3TAA increased. The addition of 3TAA resulted in an increase in the size and buildup of the polymer coating along the individual polypropylene fibers, with the largest accumulation of polymer observed with the addition of 100 mg/mL of 3TAA. The addition of 3TAA also resulted in a higher resistivity being measured for the sample, a higher elemental weight percent of sulfur in the sample, an increase in the size of the C=O stretch corresponding to the presence of the carboxylate unit and a higher fluorescent reading after the samples were put through the EDC/sulfo-NHS/FITC-avidin binding reaction. The additional carboxyl groups from the 3TAA co-polymer reacted with the EDC/sulfo-NHS cross-linking to result in this increase in the attachment of the FITC labeled avidin. However, the increase in available functional groups for additional antibody attachment came at a cost to the material’s resistivity. Increasing 3TAA changed the polymer morphology, ultimately resulting in a larger, more globular and less conductive coating. 

When developing an electrochemical biosensor, it is important that a balance be found between increasing the available binding sites for reactions to take place and decreasing the membrane resistivity to achieve maximum sensitivity. It is also important that the membrane maintain its porosity and, therefore, increased surface area, as well as being environmentally robust. In our research, an observable difference could not consistently be seen between the samples containing 0 and 1 mg/mL 3TAA. The concentration *vs*. signal outputs did show an observable difference between samples with a concentration of 10 mg/mL of 3TAA and samples without 3TAA. At a concentration of 10 mg/mL, the membranes had an average resistivity 38.3% higher than the membranes containing no 3TAA, a sulfur weight percentage 39.8% higher and an increase in average fluorescent output of 43.6%. Increases in sulfur weight percentages and fluorescence outputs when compared to the samples containing no 3TAA were also observed at concentrations of 20, 50 and 100 mg/mL, with the overall trend showing an increase in the measured value as the 3TAA concentration increased; however, all three also showed an increase in polymer buildup on the fibers, which resulted in a lower polymer durability (flaking) and higher sample resistivity. For these reasons, it was determined that the optimal concentration for use in our electrotextile biosensor assembly will be within the range of 10–50 mg/mL 3TAA.

## 4. Conclusions

A polypropylene fiber matrix was conformally coated in a conductive, functionalized polymer. Coated fiber membranes maintained porosity, surface area and coating durability, while gaining electrical conductivity and biorecognition element binding sites. The addition of 3TAA to the polymerization process resulted in a change of coating morphology, resistivity and available binding sites for biorecognition elements. Polymer coated membrane samples containing concentrations within the range of 10–50 mg/mL of 3TAA were selected as the best for future biosensor development.

Future work will be conducted to narrow the concentration range and identify the optimal concentration of 3TAA in the polymerization reaction in order to create a conductive electrotextile for use in biosensors. We also intend to explore how changes to other factors in the polymerization process, such as reaction times and the other component concentrations, will affect the conductivity and morphology of the fiber coatings. The use of a higher surface area nonwoven material may also be used to increase the number of potential binding sites within a square area. Additionally, we intend to look at applying the coating to more uniformly constructed fabrics in order to reduce variability between samples. 
